# Supplementary material for: RECQ4-MUS81 interaction contributes to telomere maintenance with implications to Rothmund-Thomson syndrome
Source: Nat Commun. 2025 Feb 3;16:1302. doi: 10.1038/s41467-025-56518-1 (PMC11791078; doi:10.1038/s41467-025-56518-1)
Supplement: Supplementary file 1 — Supplementary Information [file 41467_2025_56518_MOESM1_ESM.pdf]

Supplementary Figure 1

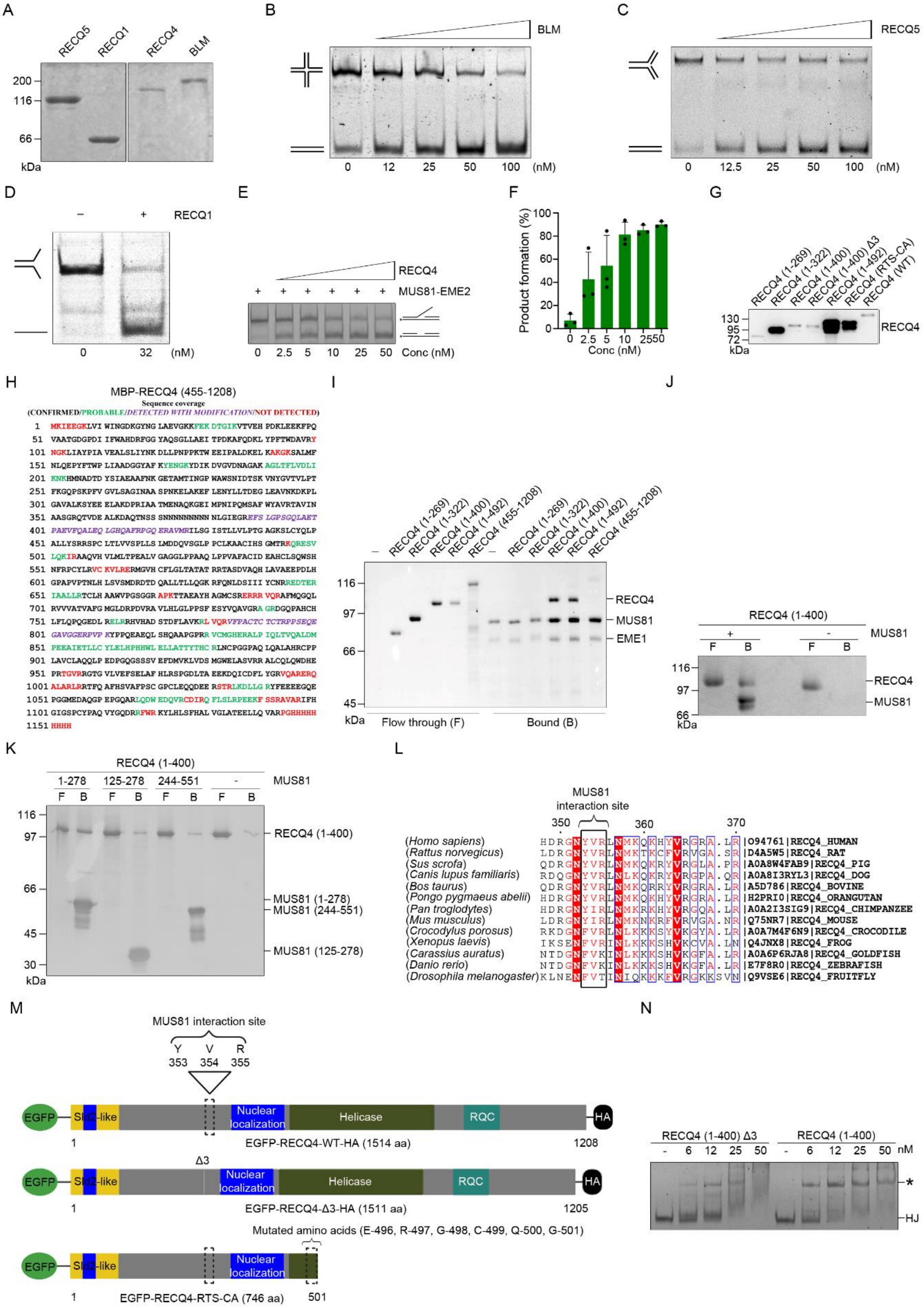

Supplementary Figure 1. RECQ4 stimulates MUS81-EME2 and interacts with MUS81 via N-terminus

(**A**) SDS-PAGE analysis of recombinant RECQ proteins used in this study (RECQ5, RECQ1, RECQ4 and BLM). (**B**) Branch migration activity of BLM protein. A mobile Holliday junction DNA substrate (6 nM) was incubated with the indicated amounts of BLM for 15 min at 37 °C in a buffer containing 25 mM Tris pH 7.5, 1 mM DTT, 0.1 mg/mL BSA, 50 mM KCl, 7.5 mM creatine phosphate, 11.25 µg/mL creatine kinase, 2.5 mM MgCl2 and 2.5 mM ATP. Reactions were stopped by the addition of SDS and proteinase K, and the samples were resolved on a 12% native PAGE. Gels were scanned using an FLA-9000 Starion image scanner (Fujifilm). The experiment was repeated at least twice. (**C**) Branch migration activity of RECQ5. A mobile fork DNA substrate (3 nM) was incubated with the indicated amounts of RECQ5 for 15 min at 37 °C in a buffer containing 25 mM Tris pH 7.5, 5 mM DTT, 0.1 mg/mL BSA, 30 mM KCl, 7.5 mM creatine phosphate, 11.25 µg/mL creatine kinase, 5 mM MgCl2 and 2.5 mM ATP. Reactions were stopped by the addition of SDS and proteinase K, and the samples were resolved on a 12% native PAGE. Gels were scanned using Molecular Imager PharosFX (Bio-Rad). The experiment was repeated at least twice. (**D**) Helicase activity of RECQ1. A Y-form DNA substrate (5 nM) was incubated with the indicated amount of RECQ1 for 20 min at 37 °C in a buffer containing 20 mM Tris pH 7.5, 5 mM DTT, 0.08 mg/mL BSA, 8% glycerol, 20 mM KCl, 5 mM MgCl2 and 5 mM ATP. Reactions were stopped by the addition of SDS and proteinase K, and the samples were resolved on a 12% native PAGE. Gels were scanned using an FLA-9000 Starion image scanner (Fujifilm). The experiment was repeated at least twice. (**E**) Increasing concentrations of RECQ4 (1-400) were incubated with a 3'-flap DNA substrate (6 nM) in the presence of MUS81-EME2 (0.2 nM) for 20 minutes. Reaction mixtures resolved on a native PAGE gel and quantified. n=3 independent experiments; data are means ± SD. (**F**) Quantification of data in (E); n=3 independent experiments; data are means ± SD. (**G**) Western blot analysis of purified RECQ4 variants used in this study: MBP-RECQ4 (1-269, 1-322, 1-400, 1-400 (Δ3), 1-492, and RTS-CA), and GST-RECQ4-WT by RECQ4 antibody. The experiment was repeated at least thrice (**H**) Mass spectrometry analysis of RECQ4 (455-1208), 92 % combined amino acids coverage was observed from MALDI-MS/MS and LC-MS/MS as depicted in the figure. Confirmed, probable, detected with modification and not detected sequences were depicted in black, green, purple, and red, respectively. (**I**) Purified MBP-RECQ4 variants (1-269, 1-322, 1-400, 1-492, and 455-1208) (each 5 µg) incubated with GST-MUS81-EME1 (5 µg) and GST-beads for 30 min at 4 °C. After incubation, the beads were washed and treated with SDS to elute bound proteins. The flow through (F) and bound (B) fractions were analysed by Coomassie blue staining. (**J**) MBP-tagged RECQ4 (1-400) (5 µg) mixed with full-length GST-MUS81 (5 µg) or GST-beads alone, followed by a 30-minute incubation at 4°C and analysed as in (B). (**K**) MBP-RECQ4 (1-400) (5 µg) incubated with various MUS81 fragments, GST-MUS81 (1-278), GST-MUS81 (125-278), and GST-MUS81 (244-551) (each 5µg), or with GST-beads alone, for 30 minutes at 4°C. After incubation, the beads were washed and treated with SDS to elute the bound proteins. The flow through (F) and bound (B) fractions were analysed by Coomassie blue staining. The experiment was repeated at least twice. (**L**) Multiple sequence alignment showing the conserved amino acid sequence of RECQ4 responsible for MUS81 interaction among indicated organisms. (**M**) Schematics of EGFP-tagged RECQ4 variants showing conserved regions, the position of the MUS81 interaction region, the position of the MUS81 interaction region, tags, patient-derived mutation with its last six amino acids mutated (ISTLLV to ERGECG), and a premature stop codon after 501 amino acids. Created in BioRender.

Ashraf, R. (2025) https://BioRender.com/e43o204 (**N**) Increasing concentrations of RECQ4 (1-400) and RECQ4 (1-400) Δ3 proteins (6, 12, 25, and 50 nM) incubated with HJ DNA substrate (3 nM) for 20 minutes. The reaction mixtures resolved on a native TBE gel. * Denotes protein/DNA complexes. The experiment was repeated at least twice. Source data are provided as source data file.

Supplementary Figure 2

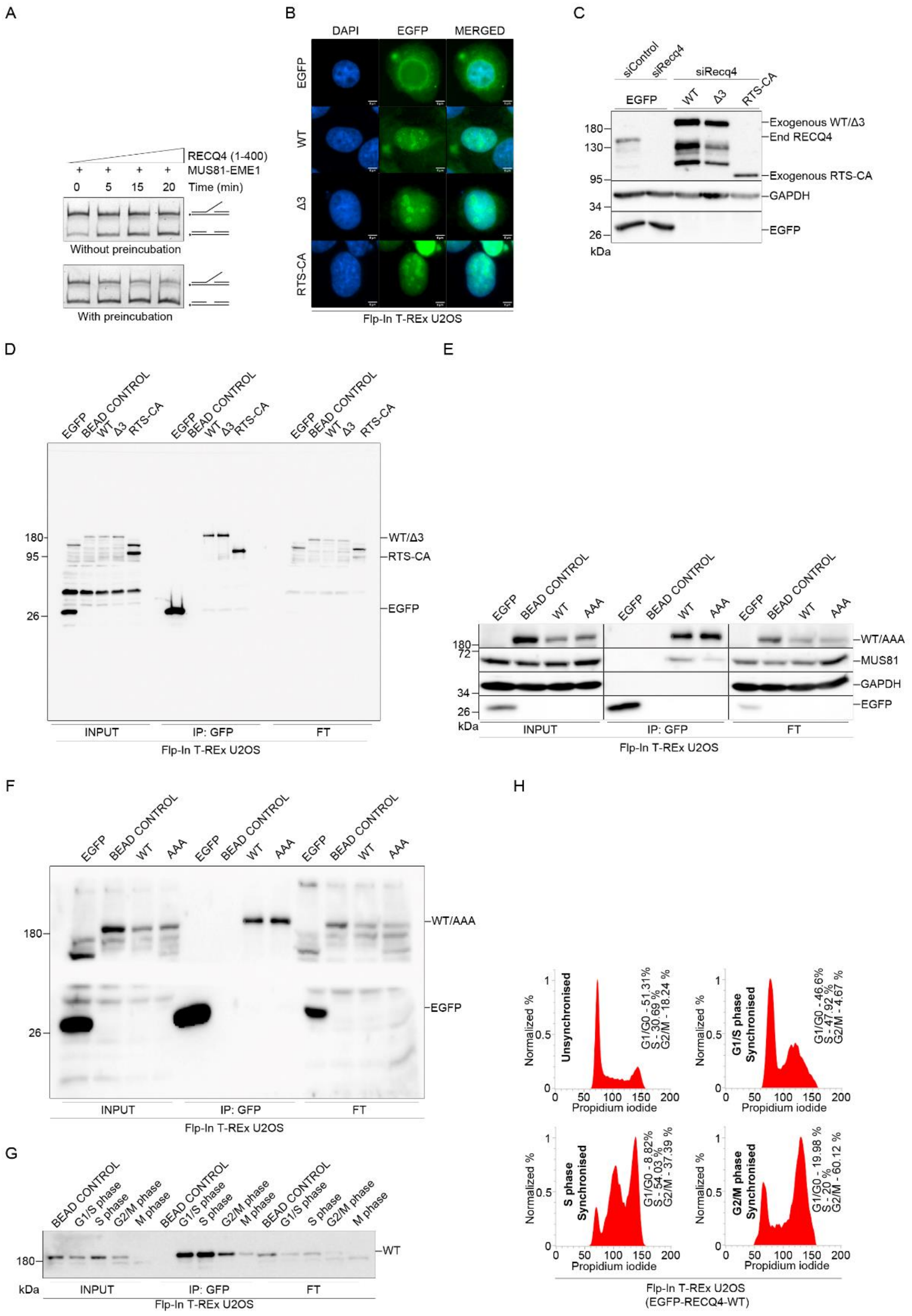

Supplementary Figure 2. Validation of interaction-deficient and patient-derived RECQ4 mutations

(**A**) Analysis of RECQ4 mediated targeting of MUS81-EME1 complex *in vitro*. RECQ4 (1-400) (25 nM) was preincubated with 3'-flap DNA substrate (6 nM) for 10 min at 37 °C, followed by the addition of MUS81-EME1 (0.5 nM) (With preincubation). Alternatively, the reaction components were mixed simultaneously (Without preincubation) with DNA and MUS81-EME1. Samples were collected at the indicated times and analysed by native gel electrophoresis. The experiment was repeated at least thrice. (**B**) Representative immunofluorescence images illustrating the subcellular localization of EGFP, EGFP-RECQ4-WT, EGFP-RECQ4-Δ3, and EGFP-RECQ4-RTS-CA. Scale bar = 5 µm. (**C**) Western blot confirming the expression of EGFP, EGFP-RECQ4-WT, EGFP-RECQ4-Δ3, and EGFP-RECQ4-RTS-CA constructs in Flp-In T-REx U2OS cells, treated with siControl or siRECQ4 to deplete endogenous RECQ4. Whole-cell extracts were separated by SDS-PAGE and analysed by western blotting detecting corresponding proteins. The experiment was repeated at least twice. (**D**) Uncropped GFP immunoblots corresponding to Figures 2A and 6D. (**E**) Whole-cell extracts (500 µg) from cells expressing EGFP, EGFP-RECQ4-WT, and the EGFP-RECQ4-AAA mutant were incubated with anti-GFP beads for one hour. The bound proteins were then separated by SDS-PAGE and analysed by western blotting detecting corresponding proteins. The experiment was repeated at least twice. (**F**) Uncropped GFP immunoblots corresponding to Figure S2E. (**G**) Uncropped GFP immunoblots corresponding to Figure 2C. (**H**) Cell cycle synchronisation profile of Flp-In T-REx U2OS cells expressing EGFP-RECQ4-WT, as determined by FACS analysis, depicting cells in unsynchronised, G1/S, S, and G2/M phases; n = 10000 cells. The experiment was repeated at least thrice. Source data are provided as source data file.

Supplementary Figure 3

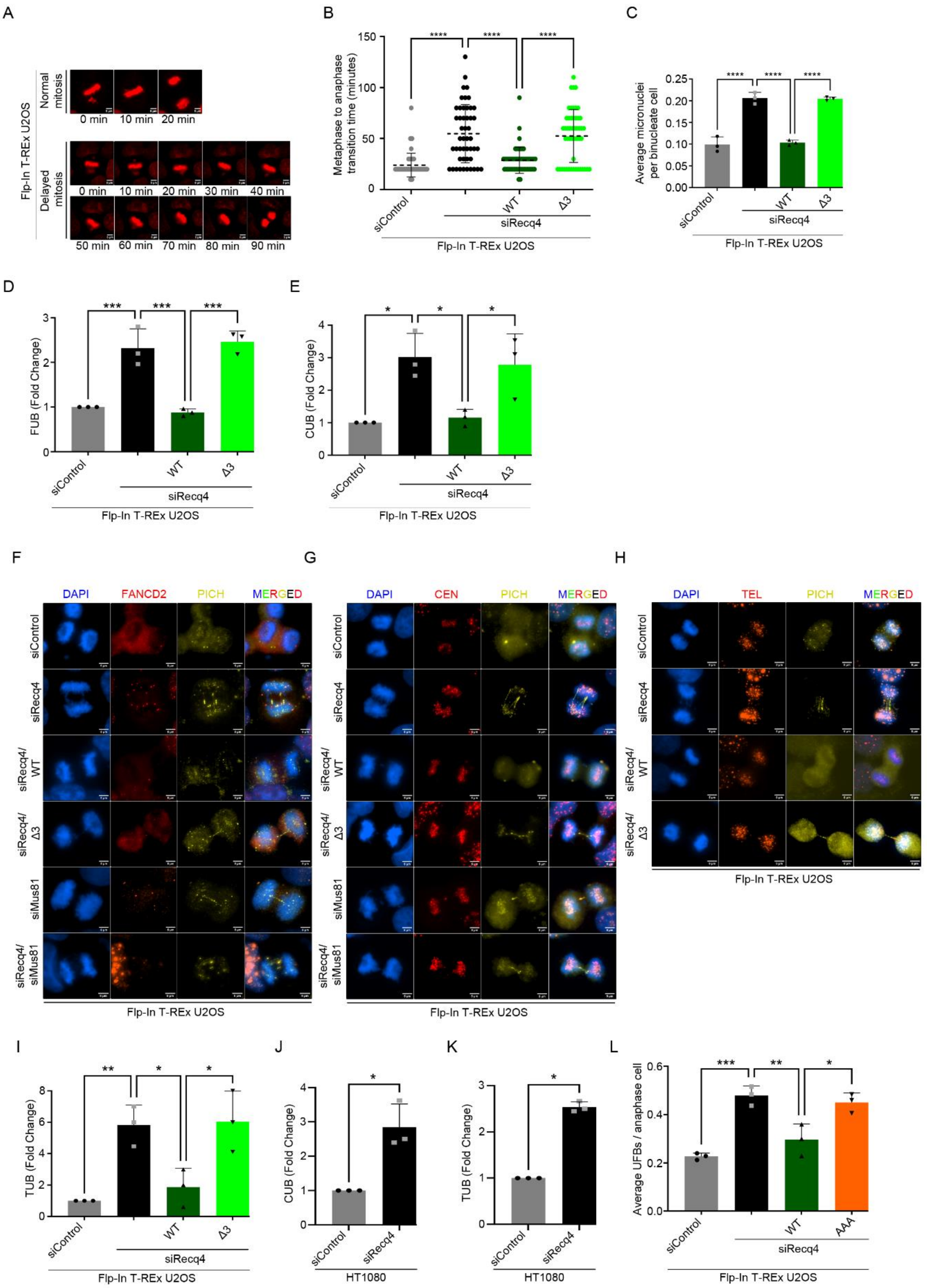

Supplementary Figure 3. RECQ4 impact on mitotic events

(**A**) Representative images depicting normal and delayed mitosis in Flp-In T-REx U2OS cells. Scale bar = 5 µm. (**B**) Quantification of the time taken for the metaphase to anaphase transition in Flp-In T-REx U2OS cells expressing either EGFP-RECQ4-WT or EGFP-RECQ4-Δ3, combined with indicated siRNA treatment. n = 45 cells; data are means ± SD; one-way ANOVA followed by Tukey's multiple comparison test; p-value were adjusted by GraphPad Prism software; siControl vs siRECQ4 ****p < 0.0001; siRecq4 vs WT/siRecq4 ****p < 0.0001; WT/siRecq4 vs Δ3/siRECQ4 ****p < 0.0001. (**C**) Quantification of the average micronuclei per binucleated cell in Flp-In T-REx U2OS cells expressing either EGFP-RECQ4-WT or EGFP-RECQ4-Δ3, combined with the indicated siRNA treatment; n=3 independent experiments; data are means ± SD; one-way ANOVA followed by Tukey's multiple comparison test; p-value were adjusted by GraphPad Prism software; siControl vs siRECQ4 ****p < 0.0001; siRecq4 vs WT/siRecq4 ****p < 0.0001; WT/siRecq4 vs Δ3/siRECQ4 ****p < 0.0001. (**D-E**) Quantification of fold change in UFBs in anaphase Flp-In T-REx U2OS cells expressing either EGFP-RECQ4-WT or EGFP-RECQ4-Δ3, combined with the indicated siRNA treatment. Depletion of endogenous RECQ4 and expression of RECQ4 constructs with doxycycline was done for 48 hours; n=3 independent experiments; data are presented as means ± SD; one-way ANOVA followed by Tukey's multiple comparison test; p-value were adjusted by GraphPad Prism software (**D**) Fragile sites ultrafine bridges (FUB); siControl vs siRECQ4 ***p = 0.0009; siRecq4 vs WT/siRecq4 ***p = 0.0005; WT/siRecq4 vs Δ3/siRECQ4 ***p = 0.0003. (**E**) Centromeric ultrafine bridges (CUB); siControl vs siRECQ4 *p = 0.0156; siRecq4 vs WT/siRecq4 *p = 0.0236; WT/siRecq4 vs Δ3/siRECQ4 *p = 0.0462. (**F-H**) Representative immunofluorescence images of different types of UFBs stained by PICH in Flp-In T-REx U2OS cells expressing either EGFP-RECQ4-WT or EGFP-RECQ4-Δ3. **(F)** Fragile sites (FUB)s was visualized by FANCD2 staining, (**G**) centromere (CUB)s by anti-centromere antibodies (CEN), and (**H**) telomeres (TUB)s by telomere PNA probe (TEL). Scale bar = 5 µm. (**I**) Quantification of fold change in telomeric ultrafine bridges (TUBs) for anaphase cells in Flp-In T-REx U2OS cells expressing either EGFP-RECQ4-WT or EGFP-RECQ4-Δ3, combined with the indicated siRNA treatment. Depletion of endogenous RECQ4 and expression of RECQ4 constructs with doxycycline was done for 48 hours; n=3 independent experiments; data are means ± SD; one-way ANOVA followed by Tukey's multiple comparison test; p-value were adjusted by GraphPad Prism software; siControl vs siRECQ4 **p = 0.0085; siRecq4 vs WT/siRecq4 *p = 0.0249; WT/siRecq4 vs Δ3/siRECQ4 *p = 0.0189. (**J-K**) Quantification of the fold change in UFBs in anaphase HT1080 cells combined with the indicated siRNA treatment. n=3 independent experiments; data are means ± SD; Mann Whitney test; one-tailed. (**J**) Centromeric ultrafine bridges (CUB); siControl vs siRECQ4 *p = 0.05. (**K**) Telomeric ultrafine bridges (TUB); siControl vs siRECQ4 *p = 0.05. (**L**) Quantification of the average number of UFBs per anaphase cell- in Flp-In T-REx U2OS cells expressing either EGFP-RECQ4-WT or EGFP-RECQ4-AAA, combined with the indicated siRNA treatment; n=3 independent experiments; data are means ± SD; one-way ANOVA followed by Tukey's multiple comparison test; p-value were adjusted by GraphPad Prism software; siControl vs siRECQ4 ***p = 0.0005; siRecq4 vs WT/siRecq4 **p = 0.0040; WT/siRecq4 vs Δ3/siRECQ4 *p = 0.0111.

Supplementary Figure 4

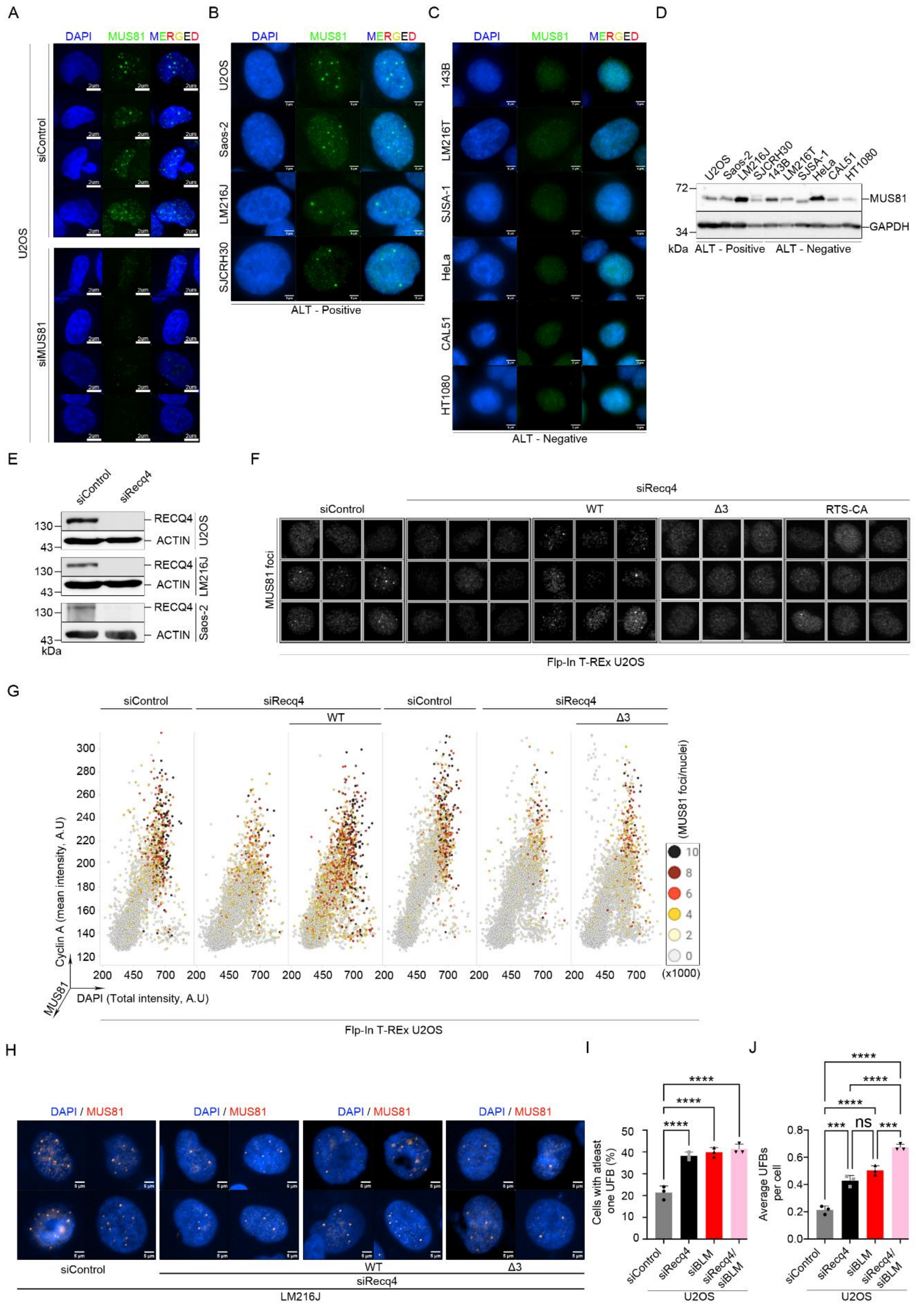

Supplementary Figure 4. RECQ4 affects MUS81 foci formation and UFB

(**A**) Representative immunofluorescence images to validate MUS81 foci in U2OS cells treated with siMUS81 and siControl for 48 hours. (**B**-**C**) Representative immunofluorescence images of MUS81 foci in a panel of ALT-positive (**B**) and ALT-negative (**C**) cell lines. (**D**) Western blot analysis confirming the presence of endogenous MUS81 in both ALT-positive and ALT-negative cell lines. Whole-cell extracts were separated by SDS-PAGE and probed with the indicated antibodies. The experiment was repeated at least twice. (**E**) Western blot confirming the effective depletion of RECQ4 in U2OS, LM216J and Saos-2 cell lines, treated with siControl or siRECQ4. Whole-cell extracts were separated by SDS-PAGE and analysed by western blotting with the indicated antibodies. The experiment was repeated at least twice. (**F**) Representative images from QIBC analysis showing MUS81 foci in Flp-In T-REx U2OS cells expressing EGFP-RECQ4-WT, EGFP-RECQ4-Δ3, and EGFP-RECQ4-RTS-CA treated with the indicated siRNA. (**G**) QIBC analysis of Flp-In T-REx U2OS cells expressing EGFP-REQ4-WT and EGFP-RECQ4-Δ3 treated with the indicated siRNA (48 hours). Cells were stained for MUS81 foci and cyclin A as cell cycle marker. Approximately 5000 cells were analysed. (**H**) Representative IF images of MUS81 foci in LM216J cells expressing EGFP-REQ4-WT and EGFP-RECQ4-Δ3 treated with the indicated siRNA. Scale bar = 5 µm. (**I**) Quantitative analysis of UFBs in U2OS cells (at least one UFB) treated with corresponding siRNA. Data are means ± SD; n=3 independent experiments; data are means ± SD; one-way ANOVA followed by Tukey's multiple comparison test; p-value were adjusted by GraphPad Prism software; siControl vs siRecq4 ****p < 0.0001; siControl vs siBLM ****p < 0.0001; siControl vs siRecq4/siBLM ****p < 0.0001. (**J**) Quantification of the average number of UFBs per anaphase cell; n=3 independent experiments; data are means ± SD; one-way ANOVA followed by Tukey's multiple comparison test; p-value were adjusted by GraphPad Prism software; siControl vs siRecq4 ***p = 0.0002; siControl vs siBLM ****p < 0.0001; siControl vs siRecq4/siBLM ****p < 0.0001; siRecq4 vs siBLM *p = 0.0195; siRecq4 vs siRecq4/siBLM **** p < 0.0001; siBLM vs sRecq4/siBLM ***p = 0.0009. Source data are provided as source data file.

Supplementary Figure 5

A

MUTATION LOCATION	MUTATION	EFFECT	CANCER TYPE	REFERENCES
N-terminus	c.1048_1049 del AG	p. Arg 350 fs X	Lymphoma	Wang *et al.*(2003), Suter *et al.* (2016), van Rij *et al.* (2017), Colombo *et al.* (2018)
N-terminus	c.1078 C > T	p. Gln 360 X	Squamous cell carcinoma, basal cell carcinoma	Suter *et al.* (2016)
N-terminus	c.1132–2 A > G	Misspiicing	Not Known	Yadav *et al.* (2019)
ZnK (Zink knuckle)	c.1222C > T	p. Gln 408 X	Not Known	Suter *et al.* (2016)
NTS (Nuclear targeting signal), MTE (mitochondrial exclusion)	c.1391-1 G > A	Misspiicing	Osteosarcoma	Lindor *et al.* (2000), van Rij *et al.* (2017)
SF2 helicase domain	c.1483 + 25 del 11	Misspiicing	Osteosarcoma	Wang *et al.* (2002), Wang *et al.* (2003)
N-terminus	c.2746-2756 del 11	Misspiicing, p. Ile 496 Glu, Ser 497 Arg, Thr 498 Gly, Leu 499 Cys, Leu 500 Gln, Val 501 Gly, Leu 501 fs X	Osteosarcoma	Balraj *et al. (2002)*

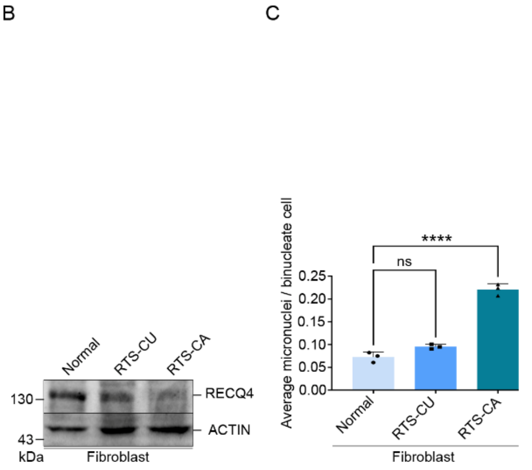

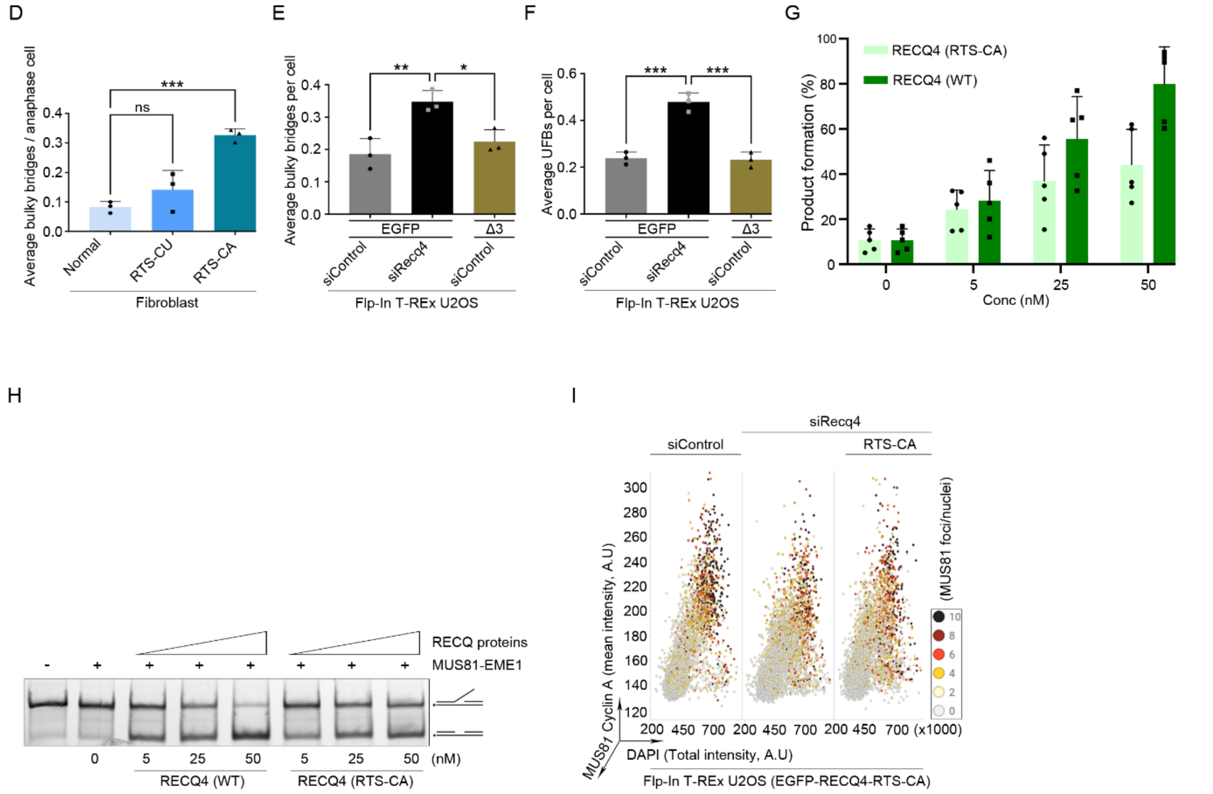

Supplementary Figure 5. Phenotypic similarities in RTS patient fibroblasts and RTS patient-derived mutants with MUS81 interaction-deficient mutant

(**A**) List of RECQ4 mutations associated with RTS that are located near the identified MUS81 interaction region. Abbreviations: (del) deletion; (>) nucleotide change from; (X) premature stop codon; (fs) frameshift mutation. (**B**) Western blot showing RECQ4 status in primary fibroblast, clinically affected (RTS-CA) and unaffected (RTS-CU) patient-derived fibroblasts.

The experiment was repeated at least twice. (**C**) Quantification of the average number of micronuclei per binucleated cell in clinically affected (RTS-CA) and unaffected (RTS-CU) patient fibroblast, along with normal fibroblast cells. Data are presented as means ± SD; n=3 independent experiments; one-way ANOVA; p-value were adjusted by GraphPad Prism software Normal vs RTS-CU ns p = 0.0588; normal vs RTS-CA ****p < 0.0001. (**D**) Quantification of the average number of bulky bridges per anaphase cell in clinically affected (RTS-CA) and unaffected (RTS-CU) patient fibroblast, along with normal fibroblast cells. Data are presented as means ± SD; n=3 independent experiments; one-way ANOVA; p-value were adjusted by GraphPad Prism software Normal vs RTS-CU ns p = 0.2293; normal vs RTS-CA ***p = 0.0007. **(E)** Quantification of the average number of bulky bridges per anaphase cell in Flp-In T-REx U2OS cells expressing endogenous RECQ4, combined with either EGFP or EGFP-RECQ4-Δ3 and treated with the indicated siRNA. EGFP/siControl (grey bar) – cells with endogenous RECQ4 and overexpression of control EGFP; EGFP/siRECQ4 (black bar) – cells with endogenous RECQ4 depleted and overexpression of control EGFP; Δ3/siControl (brown bar) – cells with endogenous RECQ4 and simultaneous overexpression EGFP-RECQ4-Δ3. Data are presented as means ± SD; n=3 independent experiments; one-way ANOVA followed by Tukey's multiple comparison test; p-value were adjusted by GraphPad Prism software; siControl vs siRecq4 **p = 0.0062; siRECQ4 vs Δ3/siControl *p = 0.0218. (**F**) Quantification of the average number UFBs per anaphase cell in Flp-In T-REx U2OS cells expressing endogenous RECQ4, combined with either EGFP or EGFP-RECQ4-Δ3 and treated with the indicated siRNA treatment. EGFP/siControl (grey bar) – cells with endogenous RECQ4 and overexpression of control EGFP; EGFP/siRECQ4 (black bar) – cells with endogenous RECQ4 depleted and overexpression of control EGFP; Δ3/siControl (brown bar) – cells with endogenous RECQ4 and simultaneous overexpression EGFP-RECQ4-Δ3. Data are presented as means ± SD; n=3 independent experiments; one-way ANOVA followed by Tukey's multiple comparison test; p-value were adjusted by GraphPad Prism software; siControl vs siRecq4 ***p = 0.0003; siRECQ4 vs Δ3/siControl ***p = 0.0002. (**G**) MUS81-EME1 (0.75 nM) and increasing amounts of RECQ4 (WT) and RECQ4 (RTS-CA) were incubated with 3'flap DNA substrate (6 nM) for 20 min at 37 °C before analysis by native gel electrophoresis; n=5 independent experiments; data are means ± SD. (**H**) Representative gel from experiment in (G). The experiment was repeated at least five times. (**I**) QIBC of Flp-In T-REx U2OS (EGFP-RECQ4-RTS-CA) cells treated with indicated siRNA and stained for MUS81 foci, DAPI and cyclin A as cell cycle marker. Approximately 5000 cells were analysed. Source data are provided as source data file.

Supplementary Figure 6

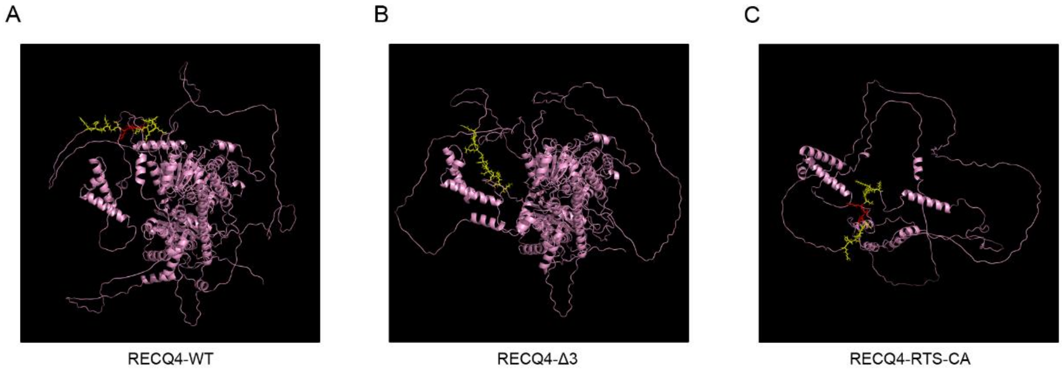

Supplementary Figure 6. AlphaFold predictions highlight folding variations in MUS81 interaction-deficient and patient-derived mutation compared to wild-type

AlphaFold protein folding prediction for different variants of the RECQ4 proteins. (**A**) RECQ4-WT, (**B**) RECQ4-Δ3, and (**C**) RECQ4-RTS-CA variant. The amino acids (YVR) responsible for MUS81 interaction are highlighted in red, surrounded by six amino acids on each side shown in yellow.
