# Supplementary material for: RECQ4-MUS81 interaction contributes to telomere maintenance with implications to Rothmund-Thomson syndrome
Source: Nat Commun. 2025 Feb 3;16:1302. doi: 10.1038/s41467-025-56518-1 (PMC11791078; doi:10.1038/s41467-025-56518-1)
Supplement: Supplementary file 2 — Reporting Summary [file 41467_2025_56518_MOESM2_ESM.pdf]

nature portfolio

Corresponding author(s): Lumir Krejci, PhD

Last updated by author(s): Dec 21, 2024

Reporting Summary

Nature Portfolio wishes to improve the reproducibility of the work that we publish. This form provides structure for consistency and transparency in reporting. For further information on Nature Portfolio policies, see our Editorial Policies and the Editorial Policy Checklist.

Statistics

For all statistical analyses, confirm that the following items are present in the figure legend, table legend, main text, or Methods section.

n/a	Confirmed	
☐	☒	The exact sample size (*n*) for each experimental group/condition, given as a discrete number and unit of measurement
☐	☒	A statement on whether measurements were taken from distinct samples or whether the same sample was measured repeatedly
☐	☒	The statistical test(s) used AND whether they are one- or two-sided<br>*Only common tests should be described solely by name; describe more complex techniques in the Methods section.*
☒	☐	A description of all covariates tested
☒	☐	A description of any assumptions or corrections, such as tests of normality and adjustment for multiple comparisons
☐	☒	A full description of the statistical parameters including central tendency (e.g. means) or other basic estimates (e.g. regression coefficient) AND variation (e.g. standard deviation) or associated estimates of uncertainty (e.g. confidence intervals)
☐	☒	For null hypothesis testing, the test statistic (e.g. *F*, *t*, *r*) with confidence intervals, effect sizes, degrees of freedom and *P* value noted<br>*Give P values as exact values whenever suitable.*
☒	☐	For Bayesian analysis, information on the choice of priors and Markov chain Monte Carlo settings
☒	☐	For hierarchical and complex designs, identification of the appropriate level for tests and full reporting of outcomes
☒	☐	Estimates of effect sizes (e.g. Cohen's *d*, Pearson's *r*), indicating how they were calculated

Our web collection on statistics for biologists contains articles on many of the points above.

Software and code

Policy information about availability of computer code

Data collection	Amersham Typhoon (for gel scanning), Luminescent Image Analyzer (for luminiscence), Nikon Eclipse Ti inverted fluorescence and Scan R inverted high-content screening microscopes (for microscopy), BD FACS verse Flow Cytometer (for FACS)
Data analysis	Graphpad PRISM 7, ImageJ, Multi Gauge, QIBC (Quantitative image-based cytometry), Spotfire, RStudio, BD FACSuite software, UCSF Chimera, T-COFFEE (Version_11.00), ESPript 3.0.

For manuscripts utilizing custom algorithms or software that are central to the research but not yet described in published literature, software must be made available to editors and reviewers. We strongly encourage code deposition in a community repository (e.g. GitHub). See the Nature Portfolio guidelines for submitting code & software for further information.

Data

Policy information about availability of data

All manuscripts must include a data availability statement. This statement should provide the following information, where applicable:

- Accession codes, unique identifiers, or web links for publicly available datasets
- A description of any restrictions on data availability
- For clinical datasets or third party data, please ensure that the statement adheres to our policy

The authors declare that all data supporting the findings of this study are available within this article, Supplementary information, or Source-data files, or from the corresponding authors upon reasonable request.

Research involving human participants, their data, or biological material

Policy information about studies with human participants or human data. See also policy information about sex, gender (identity/presentation), and sexual orientation and race, ethnicity and racism.

Reporting on sex and gender	N/A
Reporting on race, ethnicity, or other socially relevant groupings	N/A
Population characteristics	N/A
Recruitment	N/A
Ethics oversight	N/A

Note that full information on the approval of the study protocol must also be provided in the manuscript.

Field-specific reporting

Please select the one below that is the best fit for your research. If you are not sure, read the appropriate sections before making your selection.

☒ Life sciences ☐ Behavioural & social sciences ☐ Ecological, evolutionary & environmental sciences

For a reference copy of the document with all sections, see nature.com/documents/nr-reporting-summary-flat.pdf

Life sciences study design

All studies must disclose on these points even when the disclosure is negative.

Sample size	No experiments requiring sample size were used in this study.
Data exclusions	No data were excluded from this study.
Replication	For every experiment we included the number of independent replicas
Randomization	Randomization was not relevant to this study because we are not working with any particular population and our experiment can be repeated.
Blinding	Blinding was not implemented in this study as the experiments were performed without prior knowledge of the outcome.

Reporting for specific materials, systems and methods

We require information from authors about some types of materials, experimental systems and methods used in many studies. Here, indicate whether each material, system or method listed is relevant to your study. If you are not sure if a list item applies to your research, read the appropriate section before selecting a response.

Materials & experimental systems

n/a	Involved in the study	
☐	☒	Antibodies
☐	☒	Eukaryotic cell lines
☒	☐	Palaeontology and archaeology
☒	☐	Animals and other organisms
☒	☐	Clinical data
☒	☐	Dual use research of concern
☒	☐	Plants

Methods

n/a	Involved in the study	
☒	☐	ChIP-seq
☐	☒	Flow cytometry
☒	☐	MRI-based neuroimaging

Antibodies

Antibodies used	Anti-RECQ4, 1:700 dilution, 2814, CST<br>Anti-GAPDH (1:1000 dilution, 3683, CST)<br>Anti-GFP (1:2500, ab290, Abcam)<br>Anti-MUS81 (1:1000 dilution, ab14387, Abcam)<br>Anti-CENPF (1:1000 dilution, 58982S, CST)

Anti-Rabbit IgG (1:10000 dilution, A6154, Sigma-Aldrich)
Anti-Mouse IgG (1:15000, A0168, Sigma-Aldrich)
Anti-ERCC6L (1:100 dilution, H00054821-B01P, Abnova)
Anti-Centromere (1:400 dilution, HCT-0100, Immuniovision)
Anti-FANCD2 (1:500 dilution, NB100-182, Novus Biologicals)
Anti-Mouse IgG, Alexa flour 488 (1:500 dilution, A11001, Thermo Fisher Scientific)
Anti-Mouse IgG, Alexa flour 546 (1:500 dilution, A21123, Thermo Fisher Scientific)
Anti-Rabbit, Alexa flour 555 (1:500 dilution, A31572, Thermo Fisher Scientific)
Anti-Human IgG, DyLight®650 (1:500 dilution, ab98622, Abcam)
Anti-Rabbit IgG, Alexa Fluor Plus 647 (1:500 dilution, A32733, Thermo Fisher Scientific)
Anti-Mouse IgG (H+L), Alexa flour 594 (1:500 dilution, A11005, Thermo Fisher Scientific)
Anti-Mouse IgG (H+L), Alexa Fluor™ 568 (1:500 dilution, A11061, Thermo Fisher Scientific)
anti-MUS81 (1:1000, mouse, sc-53382, Santa Cruz)
anti-cyclin A (1:1000, rabbit, sc-596, Santa Cruz)
Anti-PML (1:200 dilution, sc-966, Santa Cruz Biotechnology)
Anti-Mouse IgG2a, Alexa flour 647 (1:500 dilution, A21241, Thermo Fisher Scientific)

Validation

Anti-RECQ4, 1:700 dilution, 2814, CST
validated for Western blot by the manufacturer.
Citation: PMID: 36126066, PMID: 32432680

Anti-GAPDH (1:1000 dilution, 3683, CST)
validated for Western blot by the manufacturer.
citation: PMID: 39091580, PMID: 39138527

Anti-GFP (1:2500, ab290, Abcam)
Validated for western blotting, Immuno cyto chemistry, Immunoprecipitation by the manufacturer.
Citation: PMID: 29995861, PMID: 33761359

Anti-MUS81 (1:1000 dilution, ab14387, Abcam)
Validated for western blotting, Flow cytometry by the manufacturer.
Citation: PMID: 35443178, PMID: 37105990

Anti-CENPF (1:1000 dilution, 58982S, CST)
Validated for western blotting, Flow cytometry and Immunofluorescence by the manufacturer.
Citation: PMID: 37888778, PMID: 34728620

Anti-ERCC6L (1:100 dilution, H00054821-B01P, Abnova)
Validated for western blotting by the manufacturer.
Citation: PMID: 29445165, PMID: 29235471

Anti-Centromere (1:400 dilution, HCT-0100, Immuniovision)
Validated for Immunofluorescence by the manufacturer.
Citation: PMID: 29445165, PMID: 31253795

Anti-FANCD2 (1:500 dilution, NB100-182, Novus Biologicals)
Validated for western blotting by the manufacturer.
Citation: PMID: 28445142, PMID: 36257352

anti-MUS81 (1:1000, mouse, sc-53382, Santa Cruz)
Validated for western blotting by the manufacturer.
Citation: PMID: 31803609, PMID: 36997515

anti-cyclin A (1:1000, rabbit, sc-596, Santa Cruz)
Validated for western blotting by the manufacturer.
Citation: PMID: 26644182, PMID: 16912279

Anti-PML (1:200 dilution, sc-966, Santa Cruz Biotechnology)
Validated for western blotting and Immunofluorescence by the manufacturer.
Citation: PMID: 37227756, PMID: 37059091

Eukaryotic cell lines

Policy information about cell lines and Sex and Gender in Research

Cell line source(s)

U2OS and HeLa cell line was obtained from the European Collection of Authenticated Cell Cultures (ECACC).
HT1080 cells were obtained from Dr. Soucek (Institute of Biophysics).

	Saos-2 was obtained from Dr. Uldrijan (Masaryk University).<br>SJCRH30, SJSA-1, and 143B were acquired from American Type Culture Collection (ATCC).<br>AG18373 (RTS patient fibroblast, clinically unaffected, female) and AG18371 (RTS patient fibroblast, clinically affected, male) were obtained from Coriell Institute for Medical Research.<br>Flp-In T-REx U2OS cell line was acquired from MRC PPU Reagents (University of Dundee).<br>LM216J (ALT-positive) and LM216T (ALT-negative) cell lines were obtained from Dr. Roderick J. O'Sullivan (University of Pittsburgh) and were originally described by J.P Murnane.<br>HEK-293 (GEN1 -/-) and HEK-293 (wild-type) were obtained from Dr. Stephen C. West (The Francis Crick Institute).
Authentication	The cell line was not authenticated.
Mycoplasma contamination	All was tested negative for mycoplasma contamination.
Commonly misidentified lines (See ICLAC register)	Not commonly misidentified lines were used in this study.

Flow Cytometry

Plots

Confirm that:

- [x] The axis labels state the marker and fluorochrome used (e.g. CD4-FITC).
- [x] The axis scales are clearly visible. Include numbers along axes only for bottom left plot of group (a 'group' is an analysis of identical markers).
- [x] All plots are contour plots with outliers or pseudocolor plots.
- [x] A numerical value for number of cells or percentage (with statistics) is provided.

Methodology

Sample preparation	Following cell synchronization, cells were harvested, washed with PBS, trypsinized, and fixed in 70% ethanol. The fixed cells were stored at -20 °C for further processing. Ethanol was removed by centrifugation at 1500 rpm, and the cells were washed again with PBS, discarding the supernatant. The pelleted cells were resuspended in a staining solution containing Propidium Iodide (PI, 40 µg/mL, P4170, Sigma-Aldrich), RNase A (100 µg/mL, A2760.0100, Panreac Applichem), and 0.1 % Triton X-100. The samples were incubated at 37 °C for 40 minutes and then centrifuged again. The resulting pellet was dissolved in 500 µL PBS.
Instrument	BD FACS verse Flow Cytometer (BD Biosciences).
Software	The data were analysed using BD FACSuite software V1.0.6 (BD Biosciences).
Cell population abundance	A minimum of 10,000 cells per gate were recorded for each sample.
Gating strategy	Cells were gated to exclude debris and aggregates based on forward and side scatter (FSC and SSC) profiles.

- [ ] Tick this box to confirm that a figure exemplifying the gating strategy is provided in the Supplementary Information.
