# Supplementary material for: RECQ4-MUS81 interaction contributes to telomere maintenance with implications to Rothmund-Thomson syndrome
Source: Nat Commun. 2025 Feb 3;16:1302. doi: 10.1038/s41467-025-56518-1 (PMC11791078; doi:10.1038/s41467-025-56518-1)
Supplement: Supplementary file 3 — Description of Supplementary Data files [file 41467_2025_56518_MOESM3_ESM.pdf]

Description of Additional Supplementary Files

File Name: Supplementary Data 1

Description: Oligonucleotides/ primers used in this study.
