# Supplementary material for: RECQ4-MUS81 interaction contributes to telomere maintenance with implications to Rothmund-Thomson syndrome
Source: Nat Commun. 2025 Feb 3;16:1302. doi: 10.1038/s41467-025-56518-1 (PMC11791078; doi:10.1038/s41467-025-56518-1)
Supplement: Supplementary file 4 — Supplementary Data 1 [file 41467_2025_56518_MOESM4_ESM.docx]

Supplementary data1: **Oligonucleotides/Primers**

| ***Primers for mammalian vectors*** | | |
| --- | --- | --- |
| **Oligo** | **Oligo description** | **Sequence** |
| pR2572 | GFP FOR | TGGAATTCTGCAGATATGGTGAGCAAGGGCGAG |
| pR2573 | GFP REV | CCGCAGCCGCTCCATCTCGAGATCTGAGTCCGG |
| pR2574 | RECQ4 FOR | ATGGAGCGGCTGCGGGAC |
| pR2575 | RECQ4 REV | GTCGGCGGCCTTGATGCGGGCCACCTGCAGGAG |
| shRECQ4 FOR |  | GATCTCCTAGGAAGAGCCTCATCTAAGTTCAAGAGACTTAGATGAGGCTCTTCCTATTTTTA |
| shRECQ4 REV |  | AGCTTAAAAATAGGAAGAGCCTCATCTAAGTCTCTTGAACTTAGATGAGGCTCTTCCTAGGA |
| pR3762 | Codon optimization siRECQ4 S17991 FOR | GGCCCCGCACGTAGTGTTTTTGTTTCATATTTAGCCGTACGTAATTGCCCC |
| pR3763 | Codon optimization siRECQ4 S17991 REV | GGGGCAATTACGTACGGCTAAATATGAAACAAAAACACTACGTGCGGGGCC |
| pR1781 | RECQ4 ∆353-355 FOR | CCCGCCATGACAGGGGCAATCTCAACATGAAGCAGAAACACTACGTGC |
| pR1782 | RECQ4 ∆353-355 REV | GCACGTAGTGTTTCTGCTTCATGTTGAGATTGCCCCTGTCATGGCGGG |
| pR4175 | RECQ4 RTS-CA First set FOR | CACCTGTAGGCAGCACCAGCAGCCCGCGTTCGCCAGACAGGATCCGCATGACTG |
| pR4176 | RECQ4 RTS-CA First set REV | CAGTCATGCGGATCCTGTCTGGCGAACGCGGGCTGCTGGTGCTGCCTACAGGTG |
| pR4177 | RECQ4 RTS-CA Second set FOR | CTTGCCGGCACCTGTAGGCTACCCCTGACACCCGCGTTCGCCAGACAGG |
| pR4178 | RECQ4 RTS-CA Second set REV | CCTGTCTGGCGAACGCGGGTGTCAGGGGTAGCCTACAGGTGCCGGCAAG |
| pR4250 | EGFP stop FOR | CGGCATGGACGAGCTGTACAAGTGAGGACTCAGATCT |
| pR4251 | EGFP stop REV | AGATCTGAGTCCTCACTTGTACAGCTCGTCCATGCCG |
| pR2576 | Codon optimization shRNA RECQ4 codon optimization FOR | TGGCCTCTAGGAAGAGCGTCTTCAAAAGCATCCACCCCAAAGCC |
| pR2577 | Codon optimization shRNA RECQ4 codon optimization REV | GGCTTTGGGGTGGATGCTTTTGAAGACGCTCTTCCTAGAGGCCA |
| pR5187 | Site-directed mutagenesis YVR to AAA FOR | TTTTGTTTCATATTTAGCGCTGCGGCATTGCCCCTGTCATGGCGGGCCAGC |
| pR5188 | Site-directed mutagenesis YVR to AAA REV | GGCTGGCCCGCCATGACAGGGGCAATGCCGCAGCGCTAAATATGAAACAAAA |
| ***Primers for Bacterial vectors*** | | |
| pR405 | RECQ4 fragment 1-500/600 FOR | AAGGAATTCATGGAGCGGCTGCGGGACGTGC |
| pR1013 | RECQ4 fragment 455–1208 FOR | TTGGAATTCCTGGGGCCCTCAGGGCAGTT |
| pR401 | RECQ4 fragment 500/600-1208 REV | AATGAATTCTTAGTGATGGTGATGGTGATGGTGATGGTGCCC |
| pR826 | RECQ4 fragment 1-269 REV | TTTGAATTCTTAGTGGTGGTGATGATGATGGTGATGGTGCCAGGGCTCCTCGTTCCATCT |
| pR1011 | RECQ4 fragment 1-322 REV | TTTGAATTCTTAGTGGTGGTGATGATGATGGTGATGGTGGAGTCCGTGGTACCTGGGGTT |
| pR577 | RECQ4 fragment 1–400 REV | ATTGAATTCTTAGTGGTGGTGATGATGATGGTGATGGTGCTTGGTTGTGACTGTGGCACC |
| pR705 | RECQ4 fragment 1–492 REV | CTTGAATTCTTAGTGGTGGTGATGATGATGGTGATGGTGGATCCGCATGACTGCACGCTC |
| pR5259 | RECQ4 RTS-CA First set FOR | GCAGTCATGCGGATCCTGTCTGGCGAACGCGGGCTGCTGCACCATCACCATCATCA |
| pR5258 | RECQ4 RTS-CA First set REV | TGATGATGGTGATGGTGCAGCAGCCCGCGTTCGCCAGACAGGATCCGCATGACTGC |
| pR5261 | RECQ4 RTS-CA Second set FOR | GATCCTGTCTGGCGAACGCGGGTGTCAGGGCCATCACCATCATCATCACCAC |
| pR5260 | RECQ4 RTS-CA Second set REV | GTGGTGATGATGATGGTGATGGCCCTGACACCCGCGTTCGCCAGACAGGATC |
| ***Oligonucleotides for 3’-flap DNA substrate*** | | |
| pR26 | 3′ flap DNA substrate | AATTCGTGCAGGCATGGTAGCT |
| pR27 | 3′ flap DNA substrate | AGCTACCATGCCTGCACGAATTAAGCAATTCGTAATCATGGTCATAGCT |
| pR29 | 3′ flap DNA substrate | AGCTATGACCATGATTACGAATTGCTTGGAATCCTGACGAACTGTAG |
